# Astrocytes in the External Globus Pallidus Selectively Represent Routine Formation During Repeated Reward-Seeking in Mice

**DOI:** 10.1523/ENEURO.0552-24.2025

**Published:** 2025-03-11

**Authors:** Minsu Abel Yang, Shinwoo Kang, Sa-Ik Hong, Jeyeon Lee, Nicholas L. Bormann, Sang Wan Lee, Doo-Sup Choi

**Affiliations:** ^1^ Department of Bio and Brain Engineering, Korea Advanced Institute of Science and Technology (KAIST), Daejeon 34141, Republic of Korea; ^2^Program of Brain and Cognitive Engineering, Korea Advanced Institute of Science and Technology (KAIST), Daejeon 34141, Republic of Korea; ^3^Department of Clinical Pharmacology, College of Medicine, Soonchunhyang University, Cheonan 31151, Republic of Korea; ^4^Department of Pharmacy, Pohang SM Christianity Hospital, Pohang 37816, Republic of Korea; ^5^ Departments of Radiology, Mayo Clinic College of Medicine and Science, Rochester, Minnesota 55905; ^6^Psychiatry and Psychology, Mayo Clinic College of Medicine and Science, Rochester, Minnesota 55905; ^7^ Department of Brain & Cognitive Sciences, Korea Advanced Institute of Science and Technology (KAIST), Daejeon 34141, Republic of Korea; ^8^Kim Jaechul Graduate School of AI, Korea Advanced Institute of Science and Technology (KAIST), Daejeon 34141, Republic of Korea; ^9^ Department of Molecular Pharmacology and Experimental Therapeutics, Mayo Clinic College of Medicine and Science, Rochester, Minnesota 55905; ^10^Neuroscience Program, Mayo Clinic College of Medicine and Science, Rochester, Minnesota 55905

**Keywords:** astrocyte, external globus pallidus, prototypic neuron, routine behavior, skill learning

## Abstract

The external globus pallidus (GPe) is a central part of the basal ganglia indirect pathway implicated in movement and decision-making. As a hub connecting the dorsal striatum and subthalamic nucleus (STN), the GPe guides repetitive and routine behaviors. However, it remains unknown how diverse GPe cells engage in routine formation while learning action sequences in repetitive reward-seeking conditioning. Here, in male mice, we investigated the Ca^2+^ dynamics of two GPe cell types, astrocytes and parvalbumin-expressing neurons, during routine formation. Our findings show that the dynamics of GPe astrocytes may be involved in action sequence refinement, a characteristic potentially contributing to more efficient reward-seeking behavior.

## Significance Statement

The ability to form and refine action sequences is essential for both survival and efficiency. In this study, we introduced the “routine index,” a measure that captures how consistently animals repeat a specific action sequence to maximize rewards. This tool provides a systematic approach to measure and analyze a subject's transition from varied to consistent and optimized behaviors. Furthermore, we demonstrate that Ca^2+^ dynamics of astrocytes, not neurons, within the external globus pallidus (GPe) correlate with this index, highlighting their active role in representing routine behaviors. These insights not only deepen our understanding of astrocytic functions in neural circuits but also pave the way for potential therapeutic interventions for disorders characterized by impaired decision-making and habitual behaviors.

## Introduction

Outcome-dependent action selection determines how animals adjust their relationship to the given environment. Translating separate actions into a sequence improves reliable learning (Jin et al., 2014). Animals persistently modulate each action within a sequence through trial and error until an optimal sequence is achieved (Dhawale et al., 2021; Mizes et al., 2023). This optimization process is particularly vital when efficiency is needed, such as procuring food for survival. However, our current understanding of how animals refine action sequences and the cellular processes involved is still limited.

Operant conditioning tasks, particularly a fixed ratio schedule, provide a powerful and well-controlled experimental framework for studying the refinement of action sequences in a stable environment (Jin et al., 2014; Tecuapetla et al., 2016; Geddes et al., 2018; Martiros et al., 2018; Dhawale et al., 2021; Baker et al., 2023; Kang et al., 2023). In these paradigms, animals learn to associate specific actions, such as nose-pokes, with reward delivery, allowing for the longitudinal observation of behavioral changes as learning progresses from varied exploration to consistent, efficient routines. The controlled nature of operant conditioning enables researchers to isolate learning-related changes in behavior and cellular activity, enabling investigation of the cellular mechanisms underlying routine formation.

The basal ganglia (Yin et al., 2009; Jin et al., 2014; Tecuapetla et al., 2016; Geddes et al., 2018; Martiros et al., 2018; Dhawale et al., 2021; Arber and Costa, 2022; Fang and Creed, 2023), particularly the globus pallidus externus (GPe; Aristieta et al., 2021; Baker et al., 2023; Kang et al., 2023), is critical in motor learning and refining movements. Recent studies have shown the GPe is not only just a relay nucleus (Gerfen et al., 1990) but also an integrative center that fine-tunes motor actions (Courtney et al., 2023). Furthermore, the GPe is involved in learning (Giovanniello et al., 2020; Lilascharoen et al., 2021; Mastrogiacomo et al., 2022) and integrating both sensory (Mastrogiacomo et al., 2022; Johansson and Ketzef, 2023) and reward information (Mastrogiacomo et al., 2022; Katabi et al., 2023). This new perspective places the GPe at the hub of the basal ganglia, essential for finely tuning motor and nonmotor learning.

The GPe is uniquely positioned to intermediate between inputs from the cortex, thalamus, brainstem, and dorsal striatum and outputs to the subthalamic nucleus, substantia nigra, and parafascicular nucleus of the thalamus with feedback projecting back to the dorsal striatum (Gittis et al., 2014; Courtney et al., 2023).

The GPe receives GABAergic inputs from indirect medium spiny neurons (iMSNs) from the dorsal striatum and sends the parvalbumin-expressing (PV+) GABAergic outputs to the subthalamic nuclei. Notably, the GPe contains abundant astrocyte populations (Cui et al., 2016). GPe astrocytes are therefore crucial for basal ganglia neural function, modulating synaptic activity (Kruyer et al., 2023) and neural circuit dynamics (Lines et al., 2020; Cho et al., 2022). The astrocyte contextual guidance model (Murphy-Royal et al., 2023) outlines how astrocytes adaptively regulate neural networks by releasing gliotransmitters onto neurons based on specific internal cues (Gourine et al., 2010; Murphy-Royal et al., 2020).

An animal recognizes a task's predictability, and then GPe astrocytes may stabilize action sequences by adjusting neural pathways based on consistent cues (Murphy-Royal et al., 2023). GPe astrocytes further refine the established routines, ensuring reliable behavior patterns. Thus, we hypothesize that our experimental paradigm will reveal the role of astrocytes in optimizing sequences and highlight their importance in maintaining efficient and accountable behaviors.

Here, we investigated the role of two major GPe cell types, astrocytes (GFAP+ cells) and GABAergic, PV+ cells, in forming and refining the action sequences during repetitive reward-seeking conditioning. Through an operant conditioning task, we first demonstrated that mice gradually shift from varied actions to a consistent sequence, known as a routine, for improved reward-seeking. We developed a metric, the “routine index,” to quantitatively measure routine formation. Employing the routine index, we discovered that GPe astrocytes, but not PV+ cells, represent routine formation.

## Materials and Methods

### Animals

All experimental procedures were approved by the Mayo Clinic Institutional Animal Care and Use Committee and performed following NIH guidelines. We purchased GFAP-Cre (Stock No. 024098), PV-Cre (Stock No. 017320), and DIO-GCaMP6s (Stock No. 028866) mice from The Jackson Laboratory and generated two homozygous bitransgenic (tg/tg) mice GFAP-Cre/GCaMP6s and PV-Cre/GCaMP6s in C57BL/6J background.

We used GCaMP6s, a calcium indicator known to have a high signal-to-noise ratio and sensitivity, allowing us to detect small changes in intracellular Ca^2+^ levels (Chen et al., 2013). We used the same genetically encoded calcium indicator across two cell types because different genetically encoded calcium indicators can represent the same Ca^2+^ dynamics differently due to variations in their kinetic properties and sensitivity to Ca^2+^ (Tian et al., 2012).

Mice were housed in standard Plexiglas cages. The colony room was maintained at a constant temperature (24 ± 1°C) and humidity (60 ± 2%) with a 12 h light/dark cycle (lights on at 07:00 A.M.). We used 8–10-week-old male mice for all experiments. Mice were allowed *ad libitum* access to food and water. For the operant conditioning tests, mice were food-restricted to 85% of their baseline weight, at which time they were maintained for the duration of experimental procedures.

### Stereotaxic surgery

Mice were anesthetized with isoflurane (1.5% in oxygen gas) with the VetFlo vaporizer with a single-channel anesthesia stand (Kent Scientific Corporation) and placed on the digital stereotaxic alignment system (model 1900; David Kopf Instruments). Hair was trimmed, and the skull was exposed using an 8-gauge electrosurgical skin cutter (KLS Martin). The skull was leveled using a dual tilt measurement tool. Holes were drilled in the skull at the appropriate stereotaxic coordinates. Next, we implanted an optic cannula (200/240 µm diameter, 200 µm end fiber) and fixed it into the GPe (AP −0.46 mm, ML +2.0 mm, DV −3.0 mm from bregma) of the cell type, specifically GCaMP6s-expressing mice by stereotaxic surgery. After stereotaxic surgery, we injected buprenorphine sustained-release LAB (1 mg/kg, s.c.; ZooPharm) for postsurgical pain relief.

### Immunofluorescence

Brains were fixed with 4% paraformaldehyde (Sigma-Aldrich) and transferred to 30% sucrose (Sigma-Aldrich) in phosphate-buffered saline at 4°C for 72 h. Brains were then frozen in dry ice and sectioned at 40 µm using a microtome (Leica Microsystems). Brain slices were stored at −20°C in a cryoprotectant solution containing 30% sucrose (Sigma-Aldrich) and 30% ethylene glycol (Sigma-Aldrich) in phosphate-buffered saline. Sections were incubated in 0.2% Triton X-100 (Sigma-Aldrich) and 5% bovine serum albumin in phosphate-buffered saline for 1 h, followed by incubation with the primary antibody in 5% bovine serum albumin overnight at 4°C. After three washes in phosphate-buffered saline, the sections were mounted onto a glass slide coated with gelatin and coverslipped with a VECTASHIELD antifade mounting medium (Vector Laboratories). Images were obtained using an LSM 780 laser scanning confocal microscope (Carl Zeiss). We used 488 (eGFP) and 405 channels for imaging. All antibodies were purchased from Abcam and included anti-GFAP (ab7620) and anti-parvalbumin (ab11427) for primary antibodies and anti-rabbit Alexa Fluor (ab175651) and anti-mouse Alexa Fluor (ab150113) for secondary antibodies.

### Behavioral experiments

#### Operant conditioning

**Figure 1.**
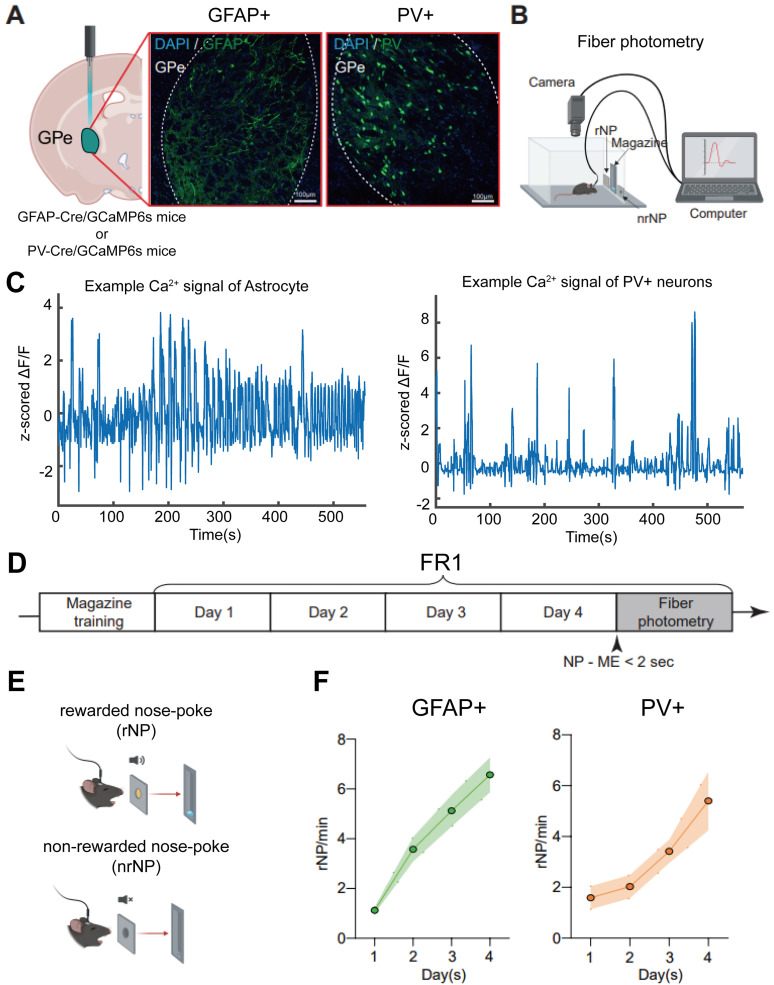
Operant conditioning task and fiber photometry. ***A***, Fiber photometry configuration to measure in vivo Ca^2+^ dynamics and histological validation of GPe astrocytes (GFAP+) and prototypic neurons (PV+). Scale, 100 µm. The images with higher magnification are presented in Extended Data Figure 1-1. ***B***, Schematic of operant conditioning task. ***C***, Example Ca^2+^ signals during the entire recording session. Left for GPe astrocytes (GFAP+). Right for prototypic neurons (PV+). The zoomed-in versions are presented in Extended Data Figure 1-2. ***D***, Schematic of the FR1 task training schedule. ***E***, Rewarded and nonrewarded nose-poke. ***F***, Rewarded nose-poking behaviors during the FR1 task training. Left for GFAP-Cre mice (*n *= 11). Right for PV-Cre mice (*n *= 10). For nonrewarded nose-poke, see Extended Data Figure 1-3. rNP/min, the number of rewarded nose-pokes per minute.

Operant chamber consisted of an active hole, an inactive hole, a magazine, a house light, a speaker, and a cue light at each nose port. Reward (20% sucrose solution) was presented to the liquid receptacle in the magazine once per reward signal by a syringe pump. Rewards are determined by nose-poking location. When mice performed a nose-poke in the active hole (rewarded or active nose-poke), the chamber presented a tone, light from the nose port, and one reward from the magazine (Fig. 1*E*, top). Conversely, when the nose-poke was performed in the inactive hole (nonrewarded or inactive nose-poke), no cues or rewards were presented (Fig. 1*E*, bottom). The operant conditioning schedule is as follows (Fig. 1*D*).

10.1523/ENEURO.0552-24.2025.f1-1 Fig 1-1 **The IHC images for histological validation and their zoomed-in version.** The top panels show GPe astrocytes (GFAP+), with green indicating GFAP and blue indicating DAPI. The bottom panels show GPe prototypic (PV+) neurons, with green indicating PV and blue indicating DAPI. The left panels display the original images (Scale: 100  µm), while the right panels show the zoomed-in versions of the corresponding images on the left (Scale: 25  µm). The red-dotted squares in the left panels depict the zoomed-in areas. Download Fig 1-1, TIF file.

**Figure 2.**
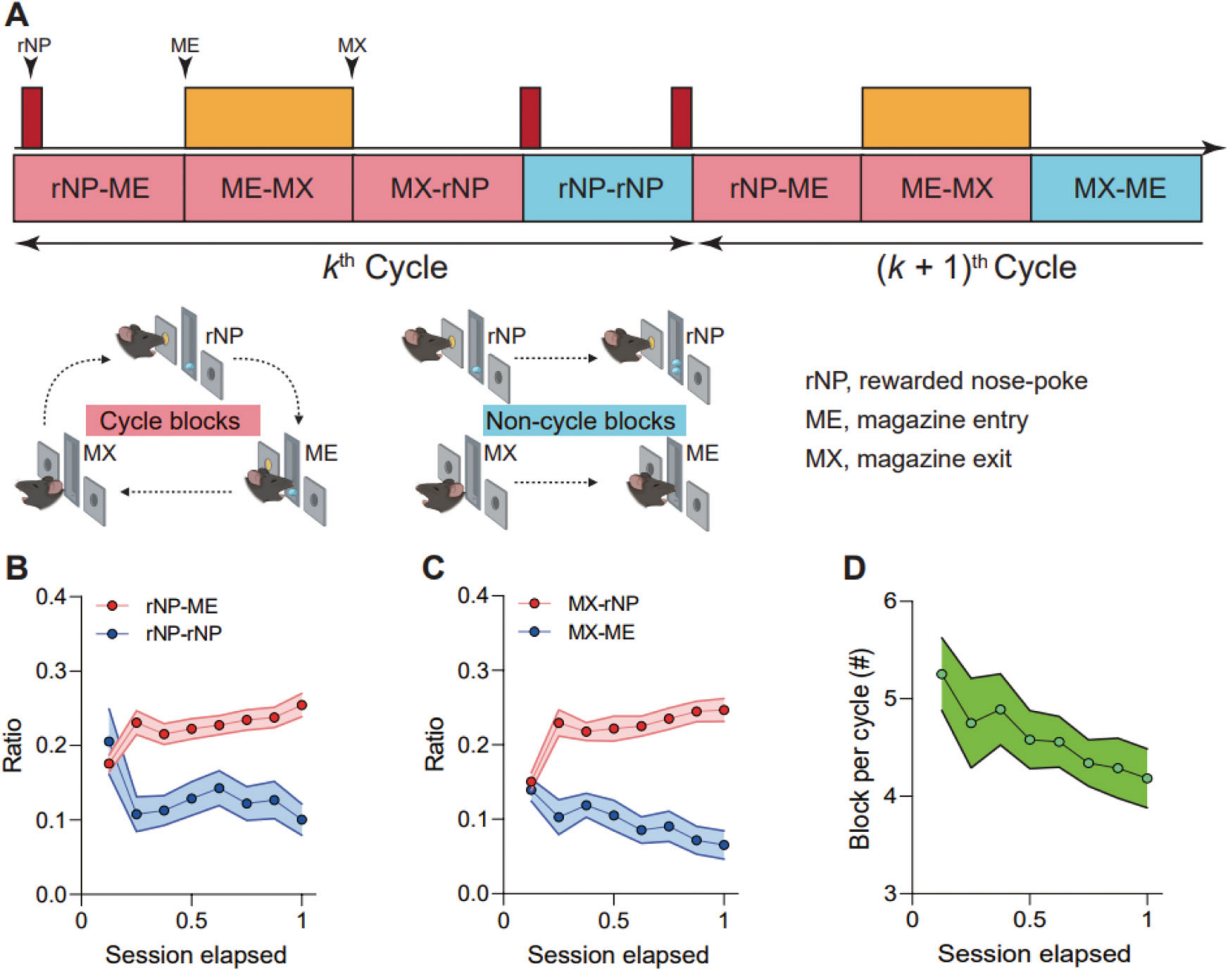
Establishment of routine behavior. ***A***, Definition of the action events, blocks, and cycle. The cycle is the sequence of blocks between rNP-ME blocks. The cycle blocks are the blocks that the cycle must include. The noncycle blocks are the blocks that do not need to finish one cycle. ***B***, ***C***, Modulation of tendency to choose the cycle block over the noncycle block after one action event. ***B***, rNP-ME over rNP-rNP after rNP (*n *= 782 blocks/rNP-ME and *n *= 410 blocks/rNP-rNP from 21 mice, including 11 GFAP-Cre mice and 10 PV-Cre mice, *p *= 0.0278 for session elapsed and *p *= 0.9345 for session elapsed × genotype). Shaded error bars denote mean ± SEM. ***C***, MX-rNP over MX-ME after MX (*n *= 772 blocks/MX-rNP and *n *= 347 blocks/MX-ME from 21 mice, including 11 GFAP-Cre mice and 10 PV-Cre mice, *p *= 8.0698 × 10^−8^ for session elapsed and *p *= 0.5141 for session elapsed × genotype). Shaded error bars denote mean ± SEM. ***D***, Block number per cycle decreases as mice spend more time inside the task environment (*n *= 782 cycles from 21 mice, including 11 GFAP-Cre mice and 10 PV-Cre mice, *p *= 0.0013 for session elapsed and *p *= 0.3015 for session elapsed × genotype). Shaded error bars denote mean ± SEM. *p < 0.05. ***B***, ***C***, Mixed-effects logistic regression with session elapsed and genotype as the fixed factor and subject as a random effect. ***D***, Mixed-effects linear regression with session elapsed and genotype as the fixed factor and subject as a random effect.

10.1523/ENEURO.0552-24.2025.f1-2 Fig 1-2 **Representative Ca^2+^ signals were recorded from GPe PV** **+** **neurons and astrocytes.** The left panels show the Ca^2+^ signal during the entire recording session. The bold black bar on the top of each graph indicates the period zoomed-in on the right panels. The right panels show the Ca^2+^ signal during the specified period on the left panels. **(A)** Example Ca^2+^ signal recorded from PV + neurons. **(B)** Example Ca^2+^ signal recorded from astrocytes. Download Fig 1-2, TIF file.

**Figure 3.**
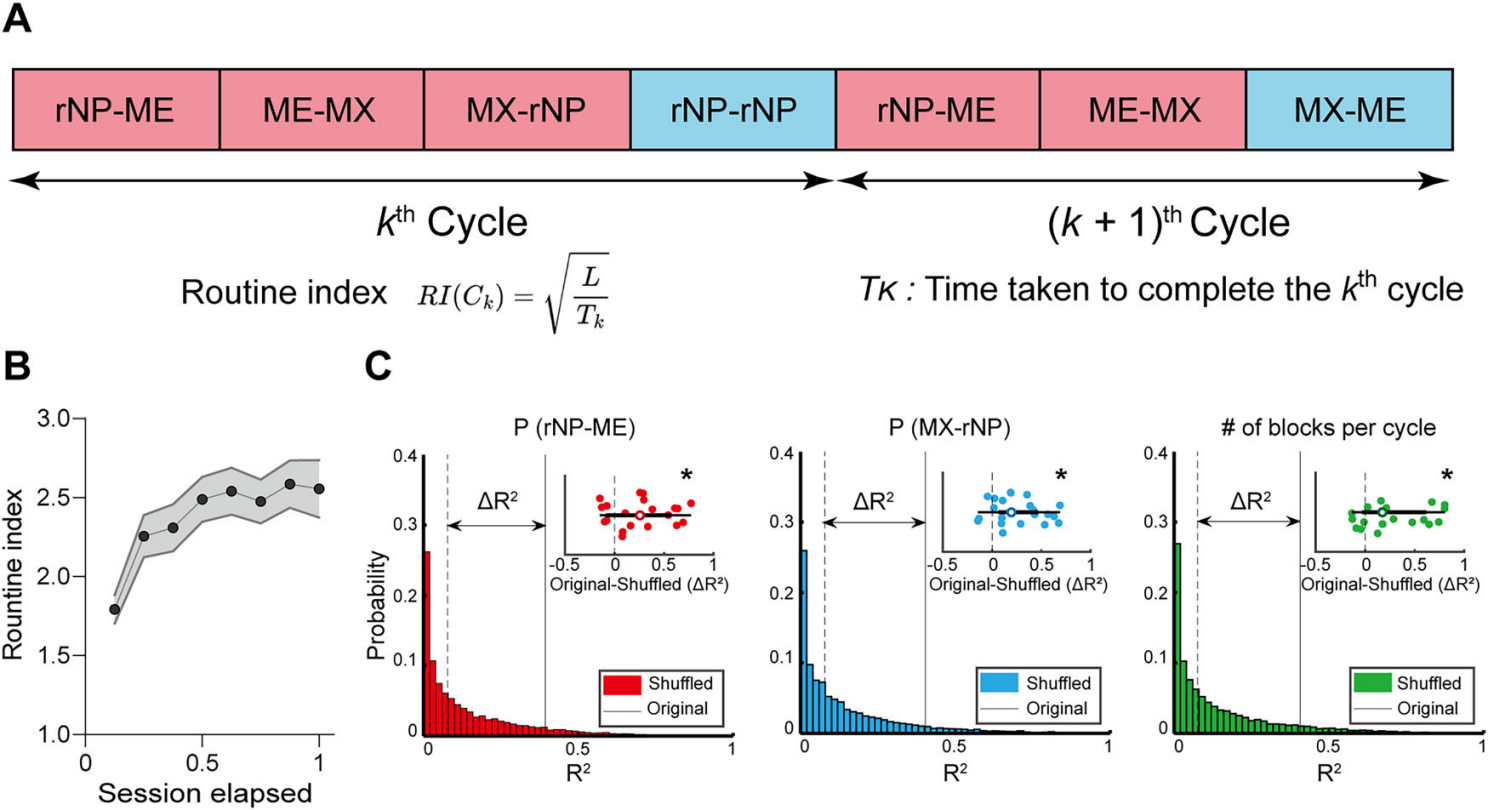
Routine index as an indicator of routine formation. ***A***, Definition of the routine index. L is a constant, the number of seconds per minutes. The transformation function of the routine index (square root function) was determined using one-sample Kolmogorov–Smirnov tests (Extended Data Fig. 3-1). ***B***, Routine index increases as mice spend more time inside the task environment (*n *= 782 cycles from 21 mice, including 11 GFAP-Cre mice and 10 PV-Cre mice, *p *= 4.6963 × 10^−8^ for session elapsed and *p *= 0.1659 for session elapsed × genotype). Shaded error bars denote mean ± SEM. ***C***, Routine index accurately captures the temporal dynamics of behavior measures characterizing routine formation (*n *= 21 mice, including 11 GFAP-Cre mice and 10 PV-Cre mice). The histogram displays the null distribution of shuffled *R*^2^ values, with the dotted line indicating the mean of this distribution. The solid line represents the mean of the original *R*^2^ values. The inset at the right top of the figure details the individual differences in *R*^2^ values between the original and shuffled conditions. Left for the proportion of rNP-ME after rNP (*p *= 6.4908 × 10^−3^ for condition and *p *= 0.5348 for condition × genotype). Middle for the proportion of MX-rNP after MX (*p *= 2.6163 × 10^−4^ for condition and *p *= 0.1093 for condition × genotype). Right for the number of blocks per cycle (*p *= 4.8785 × 10^−4^ for condition and *p *= 0.0977 for condition × genotype). Data are shown as individual dot plots and histograms. *p < 0.05. ***B***, Mixed-effects linear regression with session elapsed and genotype as the fixed factor and subject as a random effect. ***C***, Mixed-effects linear regression with condition (original vs shuffled) and genotype as the fixed factor and subject as a random effect.

10.1523/ENEURO.0552-24.2025.f1-3 Fig 1-3 **Non-rewarded nose-poke behaviors during the FR1 task training and recording sessions.** Left and middle for GFAP-Cre mice (*n* = 11) and PV-Cre mice (*n* = 10) during the training sessions, respectively. The right panel shows the non-rewarded nose-poke behaviors during the recording session. nrNP/min: the number of non-rewarded nose-pokes per minute. Download Fig 1-3, TIF file.

On Day 1, magazine training was conducted for mice to learn to obtain 60 rewards in the magazine's space. Following this, the fixed ratio of 1 (FR1) was performed for 4 session days at 60 min. Furthermore, if mice obtained 60 rewards, the session was terminated regardless of the remaining time. For rapid learning of operant behavior, 10 μl of 20% sucrose was placed in the active hole as bait before starting the FR1 session. Moreover, in two consecutive sessions, when the average latency time from nose-poke to the magazine was <2 s, no bait was placed in the active hole from the next session. In the last (fourth) session of the FR1 task, all mice performed operant behavior without prior bait presentations. The nose-poke and magazine entry time points, session duration, latency time from nose-poke to magazine approach, time spent in the magazine, and the number of nose-pokes and magazine entries were recorded using the Med-PC IV software, and the time resolution was 10 ms.

### Fiber photometry for measuring in vivo Ca^2+^ dynamics

For the fiber photometry, we included all trained mice without any exclusions. We recorded the cellular Ca^2+^ transients in real-time in vivo using fiber photometry as described previously (Baker et al., 2023). An optic fiber was linked to a patch cord, and light intensity at the fiber tip was 60 µW consistently (Baker et al., 2023; Kang et al., 2023). These output signals were projected onto a photodetector by the same optical fiber, which passed through a GFP filter. The light intensities were converted to relative fluorescence change (Δ*F*/*F*).

For the calculation of Δ*F*/*F*, we used the exponential moving average of the raw signal as a control signal (Jia et al., 2011; Mu et al., 2020; Baker et al., 2023). First, the raw fluorescence signal *F*_RAW_(*t*) was averaged to obtain *F*_AVG_(*t*) over a sliding time window of 0.75 s. Next, the control signal for a particular frame *F*_BASELINE_(*t*) was calculated as the minimum of the *F*_AVG_(*t*) in the 3 s time window preceding this frame.

Based on this control signal *F*_BASELINE_(*t*), Δ*F*/*F*(*t*) was calculated by subtracting *F*_BASELINE_(*t*) from *F*_RAW_(*t*) and then dividing by *F*_BASELINE_(*t*). Lastly, Δ*F*/*F*(*t*) was smoothed with an exponentially weighted moving window, described by a time constant *τ* of 0.2 s and a width *ω* of 1 s (Jia et al., 2011; Mu et al., 2020; Baker et al., 2023). This three-stage calculation of Δ*F*/*F* was performed using CineLyzer software (Ver. 4.4, Plexon). The Δ*F*/*F* data were then exported to MATLAB for further analyses.

The camera for observing the mice's performance and the camera for observing the fluorescence change of GCaMP6s were synchronized and recorded at 30 frames per second. In operant conditioning, we recorded Ca^2+^ dynamics in the GPe of mice during the last sessions of FR1. For preprocessing, we conducted *z*-score normalization for Ca^2+^ activity traces based on the average and standard deviation of Δ*F*/*F* computed during the entire session of each subject.

### Definition of block and cycle

To systematically dissect the behavioral sequence, we segmented the entire behavior during the session into “blocks,” defined as transitions between two adjacent action events: rewarded nose-poke (rNP), magazine entry (ME), and magazine exit (MX), resulting in five distinct blocks (Fig. 2*A*). Nonrewarded nose-pokes (nrNPs) were not included in our analysis as they were not observed during the recording session (Extended Data Fig. 1-3).

These blocks were further categorized based on their necessity for reward acquisition. Blocks essential for meeting the reward condition were termed “cycle blocks” (Fig. 2*A*, rNP-ME, ME-MX, MX-rNP), while those that were not were termed “noncycle blocks” (Fig. 2*A*, rNP-rNP, MX-ME). To analyze behavior in the context of reward acquisition, we introduced the concept of a “cycle,” defined as a sequence of blocks encapsulated by adjacent rNP-ME blocks (Fig. 2*A*).

### Definition of the routine index

To provide a real-time measure of a subject's alignment to the routine, we introduced the “routine index,” calculated for each cycle (Fig. 3*A*). The routine index is designed to be inversely proportional to the time taken to complete a given cycle, accommodating that behavior close to the routine results in quicker cycle completion. For a given cycle C, the routine index RI(C) is mathematically defined as follows:

$$\mathrm{RI}(C)=f\left(\frac{L}{T(C)}\right),$$

where L is the number of seconds per minute, T is a function that returns the time taken to complete the given cycle, and f is a transformation function introduced to normalize the distribution of the routine index, as the validity and reliability of the behavioral analyses warrant the normality of this data. In this study, the transformation function was set to the square root function.

10.1523/ENEURO.0552-24.2025.f3-1 Fig 3-1 **Comparison of Kolmogorov-Smirnov statistics across transformation functions.** Mixed-effects linear regression, with transformation function type and genotype as fixed factors and subject as a random effect, revealed a statistically significant difference in the K-S statistic between transformation function types, regardless of the genotype (*n* = 21 mice, including 11 GFAP-Cre mice and 10 PV-Cre mice, *P* = 3.6429 × 10^−9^ for transformation function type and *P* = 0.8574 for transformation function type × genotype)**.** Error bars denote mean ± SEM. Download Fig 3-1, TIF file.

The transformation function was determined based on the Kolmogorov–Smirnov (K–S) statistic, which quantifies the distance between the empirical distribution of the routine index and a normal distribution. Specifically, we applied diverse transformation functions (linear, logarithmic, reciprocal, and square root) to the routine index and measured the K–S statistic. For comparison of the K–S statistic across different transformation functions, we employed a linear mixed-effects model, followed by Dunnett's test for multiple comparisons. Extended Data Figure 3-1 depicts the result of the linear mixed-effects model analysis. Table 1 summarizes the results of Dunnett's test. Each table column contains the following information: (1) the transformation function, which is the function being compared against the square root function; (2) the Kolmogorov–Smirnov Statistic, which is the K–S statistic after applying the corresponding transformation function, provided as the mean and the standard error of the mean (SEM); and (3) the *p* value, which indicates the statistical significance of the paired samples *t* test.

**Table 1.** The results of Dunnett's test for multiple comparisons of transformation functions for normality of routine index

Transformation function	Kolmogorov–Smirnov statistic (mean ± SEM)	*p* value
Linear	0.1461 ± 0.0208 (PV+)	0.5228
	0.1268 ± 0.0092 (GFAP+)	
	0.1360 ± 0.0107 (total)	
Logarithmic	0.1623 ± 0.0099 (PV+)	0.0152
	0.1428 ± 0.0133 (GFAP+)	
	0.1521 ± 0.0083 (total)	
Reciprocal	0.2499 ± 0.0186 (PV+)	7.4585 × 10^−7^
	0.2178 ± 0.0169 (GFAP+)	
	0.2331 ± 0.0124 (total)	
Square root	0.1385 ± 0.0142 (PV+)	
	0.1264 ± 0.0106 (GFAP+)	
	0.1321 ± 0.0084 (total)	

These analyses demonstrated that the square root function normalizes the distribution of routine index most effectively. Based on these results, we chose the square root function as the transformation function for the routine index.

### Block alignment

We employed two alignment methods to visualize and analyze cellular activity within a single block type: head-fixed and tail-fixed. In head-fixed alignment, blocks were aligned to the timestamp of their initial action event, emphasizing cellular activity at the block's onset. Conversely, tail-fixed alignment aligned blocks to the timestamp of their terminal action event, focusing on cellular activity at the block's offset. The window length for both alignment methods was set to half the average block length for each block type, minimizing overlap between different alignments.

### Identifying time intervals of cellular modulation related to routine formation

**Figure 4.**
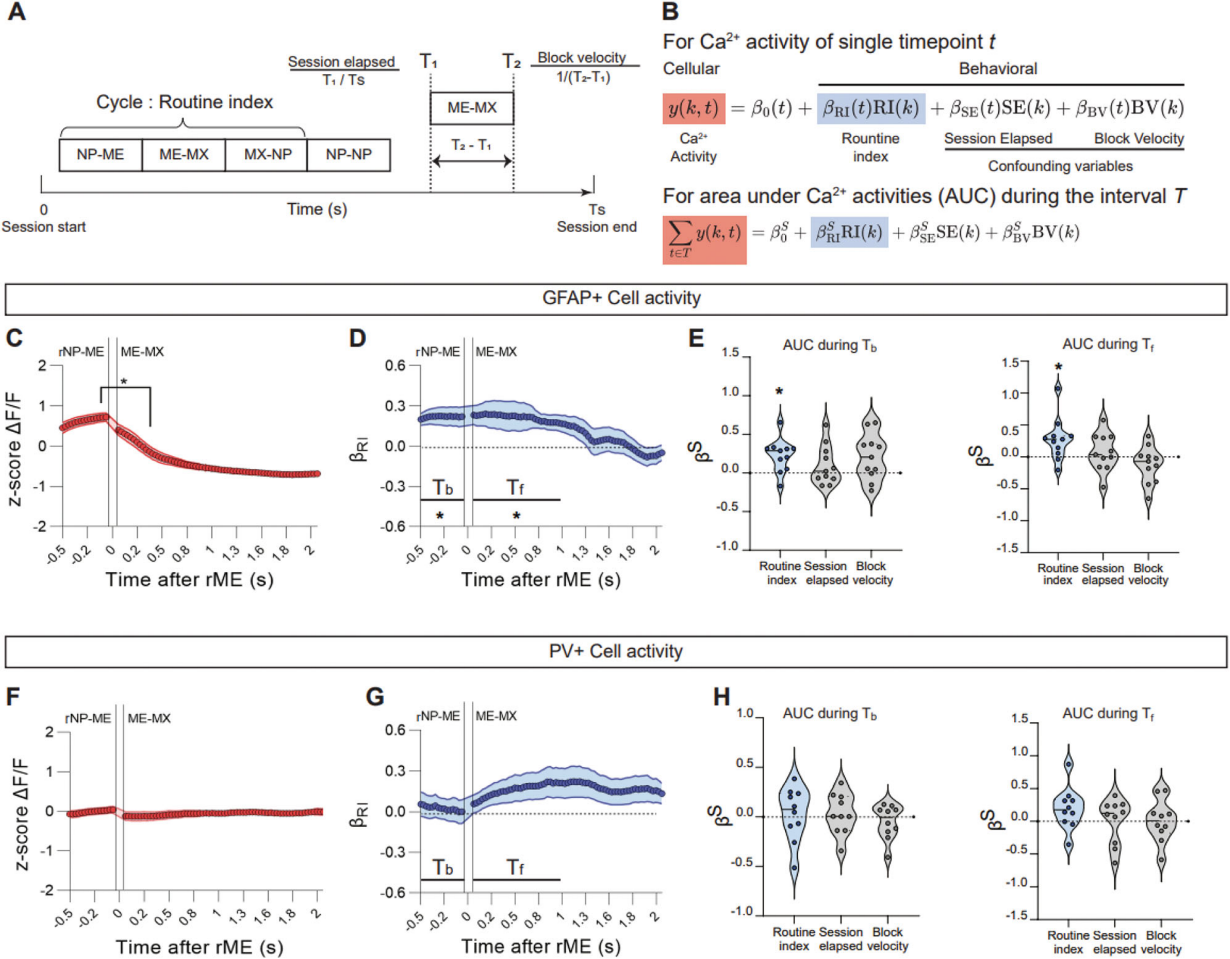
GFAP+ cell activities before and after rewarded magazine entries are associated with the degree of routine formation. ***A***, Conceptual diagram of behavioral metrics for a block. ***B***, Equations of linear regressions on cellular Ca^2+^ activity using various behavioral metrics. The validation of Ca^2+^ signal quality is detailed in Extended Data Figure 4-1. y is the matrix that contains the Ca^2+^ activity of one subject during different blocks with the same block type, which $k^{\mathrm{th}}$ row corresponds to the $k^{\mathrm{th}}$ block. RI(k), SE(k), and BV(k) are the routine index, the session elapsed, and the block velocity of the $k^{\mathrm{th}}$ block, respectively. ***C–E***, Analyses of GFAP+ cell activity (*n *= 467 blocks/rNP-ME and *n *= 442 blocks/ME-MX from 11 mice). ***C***, After the subject entered the magazine, Ca^2+^ level decreased significantly (*p *= 0.0020). The solid line and shaded regions represent mean ± SEM. Average Ca^2+^ trace of GPe astrocytes over a complete cycle (rNP → ME → MX → rNP) is presented in Extended Data Figure 4-2, top. ***D***, Regression weight of the routine index on Ca^2+^ activity before and after rewarded magazine entry. Two significant positive clusters, *T_b_* (*p *= 0.0049) and *T_f_* (*p *= 0.0044), were identified. The solid line and shaded regions represent mean ± SEM. ***E***, Regression weights on AUC during the interval *T_b_* [left, *p *= 0.0049 (routine index), *p *= 0.4648 (session elapsed), *p *= 0.0537 (block velocity)] and *T_f_* [right, *p *= 0.0098 (routine index), *p *= 0.4648 (session elapsed), *p *= 0.2061 (block velocity)]. Data are shown as individual dots and violin plots. ***F***–***H***, Analyses of PV+ cell activity (*n *= 310 blocks/rNP-ME and *n *= 281 blocks/ME-MX from 10 mice). ***F***, After rewarded magazine entry, no significant Ca^2+^ level changes were observed (*p *= 0.1309). The solid line and shaded regions represent mean ± SEM. Average Ca^2+^ trace of GPe PV+ cells over a complete cycle (rNP → ME → MX → rNP) is presented in Extended Data Figure 4-2, bottom. ***G***, Regression weight of the routine index on Ca^2+^ activity before and after rewarded magazine entry. No significant clusters were found. The solid line and shaded regions represent mean ± SEM. ***H***, Regression weights on AUC during the interval *T_b_* [left, *p *= 0.6953 (routine index), *p *= 0.4316 (session elapsed), *p *= 0.4922 (block velocity)] and *T_f_* [right, *p *= 0.1055 (routine index), *p *= 0.9219 (session elapsed), *p *= 0.9219 (block velocity)]. Data are shown as individual dots and violin plots. *p < 0.05. ***C***, ***F***, Two-sample paired Wilcoxon signed-rank test. ***D***, ***G***, Cluster-based permutation test. ***E***, ***H***, One-sample Wilcoxon signed-rank test.

Linear regressions and cluster-based permutation tests were employed to identify time intervals where the Ca^2+^ activity significantly modulates in response to the routine index. Consider a session with a duration $T_{S}$ and a block x that starts at $T_{b}$ and ends at $T_{f}$. For this block, we evaluated three behavioral metrics were evaluated: session elapsed (SE), block velocity (BV), and the routine index (RI), calculated as follows (Fig. 4*A*):

$$\mathrm{SE}(x)=\frac{T_{b}}{T_{S}},\;\;\mathrm{BV}(x)=\frac{1}{T_{f}-T_{b}},\;\;\mathrm{RI}(x)=\mathrm{RI}(C),$$

where C is the cycle to which block x belongs. For K blocks of a single type from one subject's behavioral data, a matrix $Y\in R^{K\times T}$ was constructed. Each row of Y contains the aligned Ca^2+^ activity during the corresponding block. Vectors $SE\in R^{K\times 1}$, $BV\in R^{K\times 1}$, and $RI\in R^{K\times 1}$ were also constructed, each containing the respective behavioral metrics for each block. Linear regression was then performed at each time point t as follows (Fig. 4*B*, top):

$$Y(:,t)=\beta_{0}(t)+\beta_{\mathrm{SE}}(t)SE+\beta_{\mathrm{BV}}(t)BV+\beta_{\mathrm{RI}}(t)RI,$$

10.1523/ENEURO.0552-24.2025.f4-1 Fig 4-1 **Evaluation of Ca^2+^ signal quality recorded from GPe PV** **+** **neurons and astrocytes. (A)** Ca^2+^ signals of GPe PV + neurons from an example PV-Cre mouse. Gray lines represent Ca^2+^ signals during a single rewarded magazine entry (rME). The thick red line is the average. **(B-C)** Comparison of SNR values calculated from original and shuffled Ca^2+^ signals of GPe PV + neurons (*n* = 310 blocks/rNP-ME and *n* = 281 blocks/ME-MX from 10 mice). Histograms display the null distribution of shuffled SNR values, with the dotted line indicating the mean of this distribution. The solid line represents the mean of the original SNR values. **(B)** Comparison during the rNP-ME block. **(C)** Comparison during the ME-MX block. **(D)** The individual differences in SNR values between the original and shuffled conditions for PV + neurons. **(E)** Ca^2+^ signals of GPe astrocytes from an example GFAP-Cre mouse, with the figure structure identical to **(A)**. **(F-G)** Comparison of SNR values calculated from original and shuffled Ca^2+^ signals of GPe astrocytes (*n* = 467 blocks/rNP-ME and *n* = 442 blocks/ME-MX from 11 mice), with figure structures identical to **(B-C)**. **(F)** Comparison during the rNP-ME block. **(G)** Comparison during the ME-MX block. **(H)** The individual differences in SNR values between the original and shuffled conditions for astrocytes. Download Fig 4-1, TIF file.

10.1523/ENEURO.0552-24.2025.f4-2 Fig 4-2 **Ca^2+^ trace over a complete cycle (rNP→ME→MX→rNP). (A-C)** Ca^2+^ signals of GPe astrocytes (*n* = 11 GFAP-Cre mice). **(A)** Ca^2+^ signals across block rNP-ME.(*n* = 467 blocks/rNP-ME from 11 mice). **(B)** Ca^2+^ signals across block ME-MX (*n* = 442 blocks/ME-MX from 11 mice). **(C)** Ca^2+^ signals across block MX-rNP (*n* = 376 blocks/MX-rNP from 11 mice). **(D-F)** Ca^2+^ signals of GPe PV + neurons (*n* = 10 PV-Cre mice). **(D)** Ca^2+^ signals across block rNP-ME.(*n* = 310 blocks/rNP-ME from 10 mice). **(E)** Ca^2+^ signals across block ME-MX (*n* = 281 blocks/ME-MX from 10 mice). **(F)** Ca^2+^ signals across block MX-rNP (*n* = 307 blocks/MX-rNP from 10 mice). Solid line and shaded regions represent mean ± SEM. Download Fig 4-2, TIF file.

This procedure was repeated for each of the $N_{S}$ subjects, resulting in matrices $B_{\mathrm{SE}}\in R^{N_{S}\times T}$, $B_{\mathrm{BV}}\in R^{N_{S}\times T}$, and $B_{\mathrm{RI}}\in R^{N_{S}\times T}$, which contain the regression weights for the corresponding behavior metrics from each subject. The necessity of each individual term for this analysis is detailed below.

A cluster-based permutation test was applied to $B_{\mathrm{RI}}$ to identify intervals where $\beta_{\mathrm{RI}}$ significantly deviates from 0, indicating significant modulation of Ca^2+^ activity in response to the routine index (Fig. 4*D*,*G*).

### Necessity of individual terms in the regression model

To validate the necessity of terms (Fig. 4*A*) included in our regression model (Fig. 4*B*)—specifically, routine index (RI), session elapsed (SE), and block velocity (BV)—we employed linear mixed-effects models and likelihood ratio tests. Our rationale for selecting this model and the inclusion of the interaction terms is twofold:

1. The interaction terms are essential, as the effect of independent variables (RI, SE, and BV) on calcium activity exhibits variability at different time points (Fig. 4*D*,*G*).
2. The framework of the linear mixed-effects model framework allows for the incorporation of both fixed effects (to capture population-level effects) and random effects (to account for individual differences), thus enhancing the robustness of our analysis.

Specifically, we fitted four different regression models to Ca^2+^ activity data from one cell type (either GFAP+ or PV+ cells) during specific intervals (before or after magazine entry), with the following formulas:

Model 1 (restricted model containing only RI term):

Y=RI×Time+(randomeffects|subject).

Model 2 (restricted model containing only RI and SE terms):

Y=RI×Time+SE×Time+(randomeffects|subject).

Model 3 (restricted model containing only RI and BV terms):

Y=RI×Time+BV×Time+(randomeffects|subject).

Model 4 (full model containing RI, SE, and BV terms):

Y=RI×Time+SE×Time+BV×Time+(randomeffects|subject).

We conducted likelihood ratio tests to compare the three restricted models with the full model to determine if the exclusion of any term significantly reduced the model's goodness of fit. Tables 2–5 summarize the results of these comparisons. Each column contains the necessary information for the likelihood ratio test:

1. Model: the model being compared against the full model
2. DF: the degrees of freedom, representing the number of free parameters in the regression model
3. AIC: the AIC score of the fitted regression model
4. BIC: the BIC score of the fitted regression model
5. LL: the log-likelihood of the fitted regression model
6. LR Stat: the statistics for the likelihood ratio test
7. ΔDF: the difference in the degrees of freedom between the full and restricted models
8. *p* value: the statistical significance of the likelihood ratio test

**Table 2.** The results of model comparisons to assess the necessity of SE and BV terms on Ca^2+^ activity in GFAP+ cells before magazine entry (rNP-ME)

Model	DF	AIC	BIC	LL	LR Stat	ΔDF	*p* value
RI	6	18,861	18,902	−9,424.5	433.98	4	1.261 × 10^−92^
RI + SE	8	18,740	18,795	−9,362	309.02	2	7.892 × 10^−68^
RI + BV	8	18,522	18,577	−9,252.9	90.818	2	1.902 × 10^−20^
Full	10	18,435	18,504	−9,207.5			

**Table 3.** The results of model comparisons to assess the necessity of SE and BV terms on Ca^2+^ activity in GFAP+ cells after magazine entry (ME-MX)

Model	DF	AIC	BIC	LL	LR Stat	ΔDF	*p* value
RI	6	79,564	79,614	−39,776	1,514	4	1.314 × 10^−326^
RI + SE	8	78,572	78,638	−39,278	517.35	2	4.559 × 10^−113^
RI + BV	8	78,867	78,933	−39,425	812.65	2	3.430 × 10^−177^
Full	10	78,058	78,141	−39,019			

**Table 4.** The results of model comparisons to assess the necessity of SE and BV terms on Ca^2+^ activity in PV+ cells before magazine entry (rNP-ME)

Model	DF	AIC	BIC	LL	LR Stat	ΔDF	*p* value
RI	6	13,055	13,094	−6,521.5	37.661	4	1.316 × 10^−7^
RI + SE	8	13,024	13,075	−6,503.9	2.557	2	0.278
RI + BV	8	13,059	13,110	−6,521.4	37.503	2	7.183 × 10^−9^
Full	10	13,025	13,090	−6,502.6			

**Table 5.** The results of model comparisons to assess the necessity of SE and BV terms on Ca^2+^ activity in PV+ cells after magazine entry (ME-MX)

Model	DF	AIC	BIC	LL	LR Stat	ΔDF	*p* value
RI	6	51,157	51,204	−25,573	108.89	4	1.255 × 10^−22^
RI + SE	8	51,063	51,126	−25,524	10.986	2	4.115 × 10^−3^
RI + BV	8	51,135	51,198	−25,560	83.217	2	8.505 × 10^−19^
Full	10	51,056	51,134	−25,518			

The results (Tables 2–5) demonstrated that the removal of SE and BV terms significantly impacts the model's fit across various cell types and intervals. Based on these results, we included all three terms (routine index, session elapsed, and block velocity) in our regression model.

### Evaluating the effect of behavior metrics on overall cellular activity

To assess the significance of behavioral metrics on overall Ca^2+^ activity during a prespecified interval $T_{i}$, we conducted linear regression analyses (Fig. 4*B*, bottom). For each subject, we used the matrix Y and vectors SE, BV, and RI to fit the model:

$$\underset{t\in T_{i}}{\sum }\;Y(:,t)=\beta_{0}^{S}+\beta_{\mathrm{SE}}^{S}SE+\beta_{\mathrm{BV}}^{S}BV+\beta_{\mathrm{RI}}^{S}RI.$$

This procedure was repeated for each of the $N_{S}$ subjects, resulting in vectors $\beta_{\mathrm{SE}}^{S}\in R^{N_{S}\times 1}$, $\beta_{\mathrm{BV}}^{S}\in R^{N_{S}\times 1}$, and $\beta_{\mathrm{RI}}^{S}\in R^{N_{S}\times 1}$ that contain the regression weights for the corresponding behavioral metrics. We then applied a one-sample Wilcoxon signed-rank test to these vectors to determine if the regression weights significantly differ from 0, thereby indicating a significant modulation of overall Ca^2+^ activity by the respective behavioral metrics during $T_{i}$ (Fig. 4*E*,*H*).

### Statistical analyses

All data were analyzed by one-sample Wilcoxon signed-rank test, two-sample paired Wilcoxon signed-rank test, Mann–Whitney *U* test, mixed-effects logistic regression, mixed-effects linear regression, and cluster-based permutation test using MATLAB R2023a (The MathWorks) and R (Ver. 4.1.2, R Core Team, R Foundation for Statistical Computing). The statistical significance level was set at *p *< 0.05. The number of permutations for all cluster-based permutation tests was set at *N* = 10^4^.

### Code availability

Upon publication, all codes used in this manuscript will be available at the public repository https://github.com/brain-machine-intelligence/Yang_eNeuro_2025.

## Results

### Reward-seeking operant conditioning task and measurement of Ca^2+^ dynamics using fiber photometry

To investigate the routine behavior, we employed the fixed ratio 1 (FR1) operant conditioning task. We utilized two different bitransgenic mouse lines, GFAP-Cre/DIO-GCaMP6s (*n *= 11) and PV-Cre/DIO-GCaMP6s (*n *= 10), to investigate astrocytes and neurons, respectively. Mice were trained on an FR1 schedule (one rewarded nose-poke delivers one reward, 10 μl of 20% sucrose, in the magazine) in the operant conditioning chamber (Fig. 1*B*,*D*). A single nose-poke in the active hole (rewarded nose-poke, rNP), which was accompanied by light and sound cues, enabled mice to procure a reward in the magazine (Fig. 1*E*). Mice were given 60 min or 60 rewards for each session per day. After four sessions of the FR1 task (Fig. 1*F*), we recorded Ca^2+^ signals during routine formation using fiber photometry (Fig. 1*A*,*C*). From the recorded signals, we observed that GCaMP6s effectively captures both abrupt and subtle changes in intracellular Ca^2+^ levels in GPe PV+ neurons and astrocytes (Extended Data Fig. 1-2). During the recording session, we found no significant differences between the GFAP+ and PV+ mice in the discrimination ratio (Mann–Whitney *U* test, *p *= 0.7974), the mean time spent in magazine (*p *= 0.5974), or the session duration (*p *= 0.0620).

### Routine formation in operant conditioning task

As learning progresses, we sought to determine whether mice are inclined to repeat a specific action sequence, or “routine,” for efficient reward-seeking. The routine consists of entering the active nose-poke hole, retrieving the reward in the magazine, and then returning to the active nose-poke hole (Fig. 2*A*).

To investigate the routine behavior, we assessed the changes in behavioral patterns as a function of “session elapsed,” the time since each session's start. We categorized various possible actions during task execution into three event types—rNP, magazine entry (ME), and magazine exit (MX)—and defined five blocks (Fig. 2*A*).

The analyses of block ratios over the session elapsed revealed an increase in the proportion of choosing rNP-ME block after rNP and MX-rNP block after MX, alongside a decrease in the ratio of choosing rNP-rNP block after rNP and MX-ME block after MX, irrespective of genotype (Fig. 2*B*,*C*). These findings collectively demonstrate that as time elapsed, mice gradually switched reward-seeking behaviors from variable actions to a consistent routine (rNP → ME-MX → rNP, Fig. 2*A*).

Given this shift toward repeating the routine, the mice would pass fewer blocks between successive rewarded magazine entries (rNP-ME block). To assess these behavior patterns, we examined the number of blocks per cycle (the sequence of blocks between rNP-ME blocks, Fig. 2*A*) and confirmed a decrease as the session elapsed across all genotypes (Fig. 2*D*).

Next, we quantified the alignment between each subject's current behavior and the acquired routine for systematic computation. Accommodating the view that settling into the routine results in quicker cycle completion, we propose a “routine index” (Fig. 3*A*). The routine index RI of the cycle C is defined as follows:

$$\mathrm{RI}(C)=\sqrt{\frac{L}{T(C)}},$$

where T(C) is the time taken to complete the cycle C and L is a constant denoting the number of seconds per minute.

RI(C) increases as the mice concentrate on completing the cycle (C) of rNP → ME-MX → rNP, the optimal action sequence for reward maximization. It was found to increase as the session continued regardless of genotype (Fig. 3*B*).

To validate the routine index's ability to accurately reflect routine formation, for each subject, we calculated coefficients of determination (*R*^2^) between the temporal dynamics of the routine index and behavioral measures over the session elapsed (Fig. 3*C*, original condition). These measures included the proportion of rNP-ME after rNP (Fig. 2*B*), the proportion of MX-rNP after MX (Fig. 2*C*), and the number of blocks per cycle (Fig. 2*D*). We then randomized the data across subjects and recalculated the *R*^2^ values, creating a null distribution (Fig. 3*C*, shuffled condition). Comparing the *R*^2^ between the original data and the shuffled samples revealed significant correlations between the routine index and all behavioral measures (Fig. 3*C*).

Our novel analysis revealed that mice progressively shift from various actions to a consistent routine in their reward-seeking behavior over time. The proposed routine index enabled effective characterization of the routineness of the animal's current behavior.

### GPe astrocytes encode the routine formation

After establishing the routine index, we then decided to investigate whether GPe astrocytes or PV+ neuron activities are associated with routine formation.

To assess the quality of the Ca^2+^ signal recorded from GPe PV+ neurons (Extended Data Fig. 4-1*A*) and astrocytes (Extended Data Fig. 4-1*E*), we computed the signal-to-noise ratio (SNR) of the Ca^2+^ signal around the magazine entry.

To evaluate its significance, we randomized the data across time points and recalculated the SNR values, creating a null distribution (Extended Data Fig. 4-1*B*,*C*,*F*,*G*, shuffled condition). Comparing the SNR between the original data and the shuffled samples, we found that the original SNR values of Ca^2+^ signals were significantly higher than those from the null distribution for both PV+ neurons (Extended Data Fig. 4-1*D*) and astrocytes (Extended Data Fig. 4-1*H*).

To precisely test the association between routine formation and the change in Ca^2+^ activity by accounting for various behavioral confounders, we considered additional metrics for each block: “session elapsed” ( SE, time since session start) and “block velocity” ( BV, speed of block completion), as detailed in Figure 4*A*.

We then conducted linear regression analyses on Ca^2+^ activity at individual time points for each block type and subject using the session elapsed, block velocity, and routine index as dependent variables (Fig. 4*B*, top). We sought to identify the interval where the cellular activities significantly correlate with the routineness of the animal's status quo behavior.

We analyzed the Ca^2+^ activity of GFAP+ and PV+ cells separately to investigate their respective roles in representing the progressing routine formation. A comparison of average Ca^2+^ activity just before (from −0.5 to 0 s) and just after (from 0 to 0.5 s) magazine entry revealed that the Ca^2+^ signals of GFAP+ cells were substantially decreased after magazine entry (Fig. 4*C*).

From *β*_RI_, we identified that Ca^2+^ activity before (*T_b_*: from −0.5 to 0 s) and after (*T_f_*: from 0 to 1.1 s) rewarded magazine entry increases as the routine formation progresses (Fig. 4*D*).

Post hoc analyses (Fig. 4*B*, bottom) on the area under the curve (AUC) during the identified intervals (Fig. 4*D*, bottom) revealed positive effects for the routine index in both clusters (Fig. 4*D*,*E*). Both session elapsed and block velocity exhibited no significant effects (Fig. 4*E*).

In contrast, PV+ cells showed no significant Ca^2+^ level changes after rewarded magazine entry (Fig. 4*F*). *β*_RI_ analyses found no significant clusters (Fig. 4*G*). As expected, linear regressions on the AUC during *T_b_* and *T_f_* revealed no significant impact from any behavioral metrics on PV+ cell activity (Fig. 4*H*).

## Discussion

Our study reveals that GPe astrocyte patterns coincide with routine formation in repetitive reward-seeking behaviors during an operant conditioning task. We investigated the Ca^2+^ dynamics of GPe astrocytes and prototypic neurons, revealing that a “routine” emerges with training and is selectively represented by GPe astrocytes. As mice accrued experience in the operant task, their initially variable actions transitioned into a consistent and efficient routine for reward procurement. We quantified this behavioral shift using a novel “routine index,” which correlated significantly with a spectrum of behavioral measures, validating its efficacy in tracking routine establishment.

Building upon prior research showing routine formation across diverse tasks with extended training (Tecuapetla et al., 2016; Geddes et al., 2018; Martiros et al., 2018; Shamash et al., 2021) and associated activity changes in various brain regions or cell types (Yin et al., 2009; Tecuapetla et al., 2016; Geddes et al., 2018; Martiros et al., 2018; Vandaele et al., 2019; Aristieta et al., 2021), our findings highlight the GPe as an important region in the routinization process of reward-taking behavior. Furthermore, considering our previous work showing GPe astrocyte involvement in the habit/goal-directed transition (Kang et al., 2023), the current results suggest a broader role for these cells in behavioral modulation beyond this dichotomy. Indeed, given the recognized similarity between skills and habits (Du et al., 2022), our data support the view that skill learning and routine formation are not easily categorized within a simple habit/goal-directed framework (Du et al., 2022), emphasizing the complex role of GPe astrocytes.

Based on our observations (Fig. 4), we propose a mechanism for astrocytic representation of routine formation centered on GABA signaling. The GPe microenvironment is enriched with GABA, primarily due to the inhibitory input from the striatal indirect pathway (Courtney et al., 2023). Notably, striatal indirect pathway neurons reduce the firing rate upon reward delivery (Shin et al., 2018; Martinez et al., 2022), a temporal pattern that closely aligns with our findings. This suggests that GPe astrocytes integrate GABAergic information from upstream striatal neurons to represent routine formation.

Among GABA-related processes, we posit that GABA transporter type 3 (GAT3) is a key mediator of this astrocytic representation. GAT3 is known to regulate GPe astrocyte activity (Boddum et al., 2016; Chazalon et al., 2018; Kang et al., 2023). Specifically, in response to increased GABA levels, GAT3 triggers a rise in intracellular Na^+^ concentrations, which leads to increasing Ca^2+^ levels via the Na^+^/Ca^2+^ exchange (Boddum et al., 2016). This mechanism allows astrocytes to sense and reflect ambient GABA levels in their Ca^2+^ signals. It also explains the observed decrease in astrocytic Ca^2+^ levels following the rewarded magazine entry, as striatal GABA input diminishes postreward (Shin et al., 2018). The astrocyte-specific expression of GAT3 (Conti et al., 2004; Scimemi, 2014) further provides a compelling explanation for the cell-type specificity observed in our study. Consistent with the repetitive nature of routine formation and its parallels to skill learning and habit development (Du et al., 2022), both repetitive behaviors in general (Yu et al., 2018) and habitual behavior specifically (Kang et al., 2023) are known to upregulate GAT3 expression. Therefore, we propose that GAT3 expression may similarly increase during routine formation, leading to enhanced astrocyte sensitivity to GABA and amplified calcium signaling. This proposed mechanism is consistent with our observation of the progressively increasing Ca^2+^ levels in GPe astrocytes as routines become established.

Having proposed a GAT3-based mechanism for astrocyte representation of routine formation, we now consider how these findings broaden our understanding of astrocyte function in cognition beyond the habit/goal-directed dichotomy. A recent study demonstrated hippocampal astrocyte encoding of reward location after task familiarization (Doron et al., 2022). Our study expands this to a continuous scale, the routine index, suggesting astrocyte involvement in monitoring and evaluating action sequences based on internal and external states. This economical behavior facilitates cognitive reserve (Du et al., 2022), aligning with the “contextual guidance model” of astrocyte function (Murphy-Royal et al., 2023). In this framework, astrocytes adaptively modulate neural networks by fine-tuning modulatory signal intensity, preventing maladaptive neural changes.

While these findings highlight a role for GPe astrocytes in routine formation, several limitations naturally lead to crucial future research directions. The correlational nature of our study is a primary consideration, necessitating direct investigations into causality. To address this, future studies should employ techniques like closed-loop optogenetics (Grosenick et al., 2015; Srinivasan et al., 2018). By manipulating GPe astrocyte activity during key phases such as magazine entry, we can directly test their causal impact on the development of routine. Furthermore, the proposed GAT3 mechanism requires direct validation. Future research must experimentally modulate GAT3 activity in GPe astrocytes to determine its causal role in routine formation and astrocytic signaling.

Another important avenue for future study stems from our focus on male mice. In the current study, we focused on male mice to ensure consistency with previous operant conditioning paradigms (Jin et al., 2014; Tecuapetla et al., 2016; Baker et al., 2023; Kang et al., 2023) and to minimize variability from female specific-physiological processes like the estrous cycle. Recognizing that astrocytic physiology, gene expression, and Ca^2+^ activity can differ between sexes (Johnson et al., 2008; Yagi and Galea, 2019), examining female cohorts is an important next step, as it could uncover sex-specific astrocyte mechanisms. Future studies validating whether similar astrocytic signaling dynamics are observed in females are necessary.

Beyond causality and sex differences, our focus on GFAP-expressing astrocytes opens the door to exploring the diversity of astrocytic subtypes within the GPe (Kang et al., 2020; Lee et al., 2020; Hasegawa et al., 2022; Mastrogiacomo et al., 2022; Serra et al., 2022; de Ceglia et al., 2023). Future studies utilizing additional markers such as S100 calcium-binding protein B (S100β), aquaporin-4 (AQP-4), and aldehyde dehydrogenase 1 family member L1 (ALDH1L1), can systematically compare astrocyte subtypes and analyze their respective roles during routine development.

Beyond these considerations of cell type, methodological advancements in imaging are also vital for future progress. While our study utilized GCaMP6s, a valuable tool that has significantly advanced our understanding of Ca^2+^ dynamics in various cell types (Chen et al., 2013; Dana et al., 2014; Cox et al., 2016; Bovetti et al., 2017; Randi et al., 2023), including GPe PV+ neurons (Lilascharoen et al., 2021) and astrocytes (Kang et al., 2023), we recognize that our study is limited by the lack of subcellular and network-level imaging. Future studies employing more advanced imaging techniques (Zhang et al., 2023) will be crucial to overcome these limitations and gain a more comprehensive understanding. Specifically, to dissect the cooperative dynamics between GPe neurons and astrocytes in shaping routine behavior over extended training, future research should incorporate simultaneous monitoring of cellular networks (Serra et al., 2022) and long-term in vivo imaging (Zhang et al., 2022). Furthermore, to move beyond whole-cell calcium measurements and explore the spatial complexity of astrocyte signaling, targeted recordings focused on specific subcellular compartments (Institoris et al., 2022) are needed. This is particularly relevant given the evidence that astrocyte calcium fluctuations are not limited to small processes (Grosche et al., 1999) but occur in various compartments (Bazargani and Attwell, 2016; Semyanov et al., 2020), including the soma (Mehina et al., 2017; Lines et al., 2020; Miguel-Quesada et al., 2023; Rupprecht et al., 2024), processes, and end-feet (Mulligan and MacVicar, 2004; Girouard et al., 2010; Tran et al., 2018; Institoris et al., 2022), each with potentially distinct functional roles (Mehina et al., 2017; Stobart et al., 2018; Lines et al., 2020). Finally, to fully elucidate the complexity of astrocyte function (Bazargani and Attwell, 2016) within GPe circuits, future investigations should employ imaging techniques capable of resolving signal transduction pathways (Lao-Peregrin et al., 2024), allowing for the evaluation of specific signaling mechanisms (Kang et al., 2023) and their downstream consequences on neuronal activity and behavior within the context of routine formation. These advanced imaging approaches will be essential to fully unravel the intricate roles of GPe astrocytes in shaping behavioral routines.

In conclusion, this study casts new light on the cellular dynamics that facilitate the development of behavioral routines, underscoring the significant function of GPe astrocytes within this context. Our findings enhance the current understanding of the brain's adaptability during repeated reward-seeking behavior and establish a foundational platform for subsequent studies into the neural intricacies of behavioral routines.

## Data Availability

All data are available from the authors upon reasonable request.

## Synthesis

Reviewing Editor: Sam Golden, The University of Washington Decisions are customarily a result of the Reviewing Editor and the peer reviewers coming together and discussing their recommendations until a consensus is reached. When revisions are invited, a fact-based synthesis statement explaining their decision and outlining what is needed to prepare a revision will be listed below. The following reviewer(s) agreed to reveal their identity: Francesco Papaleo.

Dear Dr. Lee,

Your manuscript has been reviewed by the same two expert reviewers, who feel the manuscript is appropriate for publication.

Reviewer 2 did provide the following comment, which you can address in the discussion section and add during proofing. Please consider adding a sentence or two to discuss the lack of GFP control.

Congratulations on your manuscript moving forward.

Reviewer 2 Comment:

The authors addressed all my previous points. However, related to the comment on Figure 4 and the decrease in astrocytes activity. The authors argued that

"With raw Ca2+ signal from GPe astrocytes (Figure R3E), these SNR analysis results provided evidence suggesting that the observed decrease in GPe astrocyte activity after rewarded magazine entry is unlikely to be due to an artifact."

However, we have data (unpublished) performed in a similar operant task indicating that indeed that decrease is an artifact. This is why I was asking and suggesting using GFP recordings as control. It would be much better for the authors to be sure about their own data. This is my suggestion of course, but I would highly recommend in the interest of the authors.
