# Supplementary figures and images for: Astrocytes in the External Globus Pallidus Selectively Represent Routine Formation During Repeated Reward-Seeking in Mice
Source: eNeuro. 2025 Mar 11;12(3):ENEURO.0552-24.2025. doi: 10.1523/ENEURO.0552-24.2025 (PMC11913404; doi:10.1523/ENEURO.0552-24.2025)

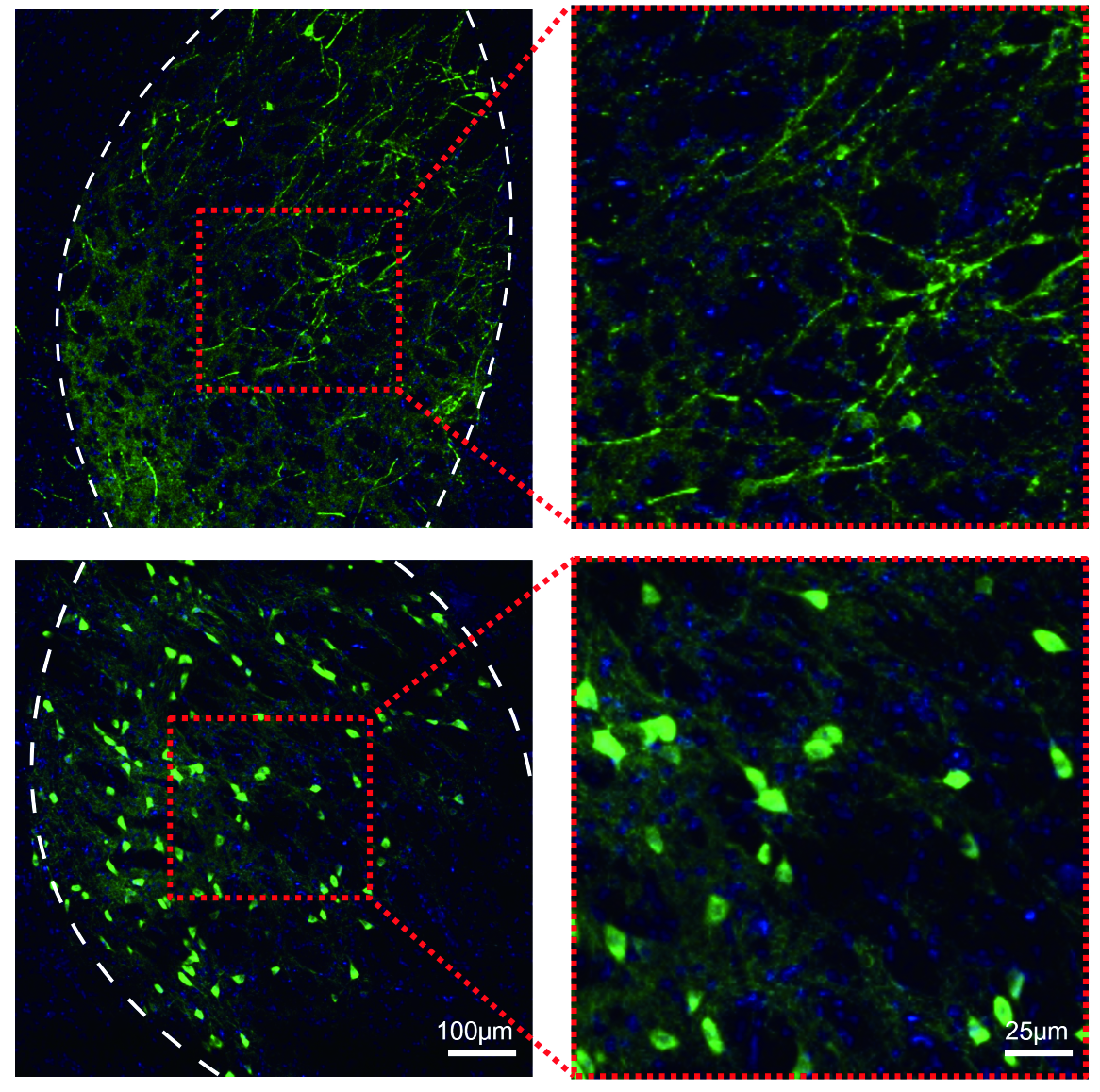

Supplement: Fig 1-1 — The IHC images for histological validation and their zoomed-in version. The top panels show GPe astrocytes (GFAP+), with green indicating GFAP and blue indicating DAPI. The bottom panels show GPe prototypic (PV+) neurons, with green indicating PV and blue indicating DAPI. The left panels display the original images (Scale: 100 µm), while the right panels show the zoomed-in versions of the corresponding images on the left (Scale: 25 µm). The red-dotted squares in the left panels depict the zoomed-in areas. Download Fig 1-1, TIF file. [file eneuro-12-ENEURO.0552-24.2025-s002.tif]

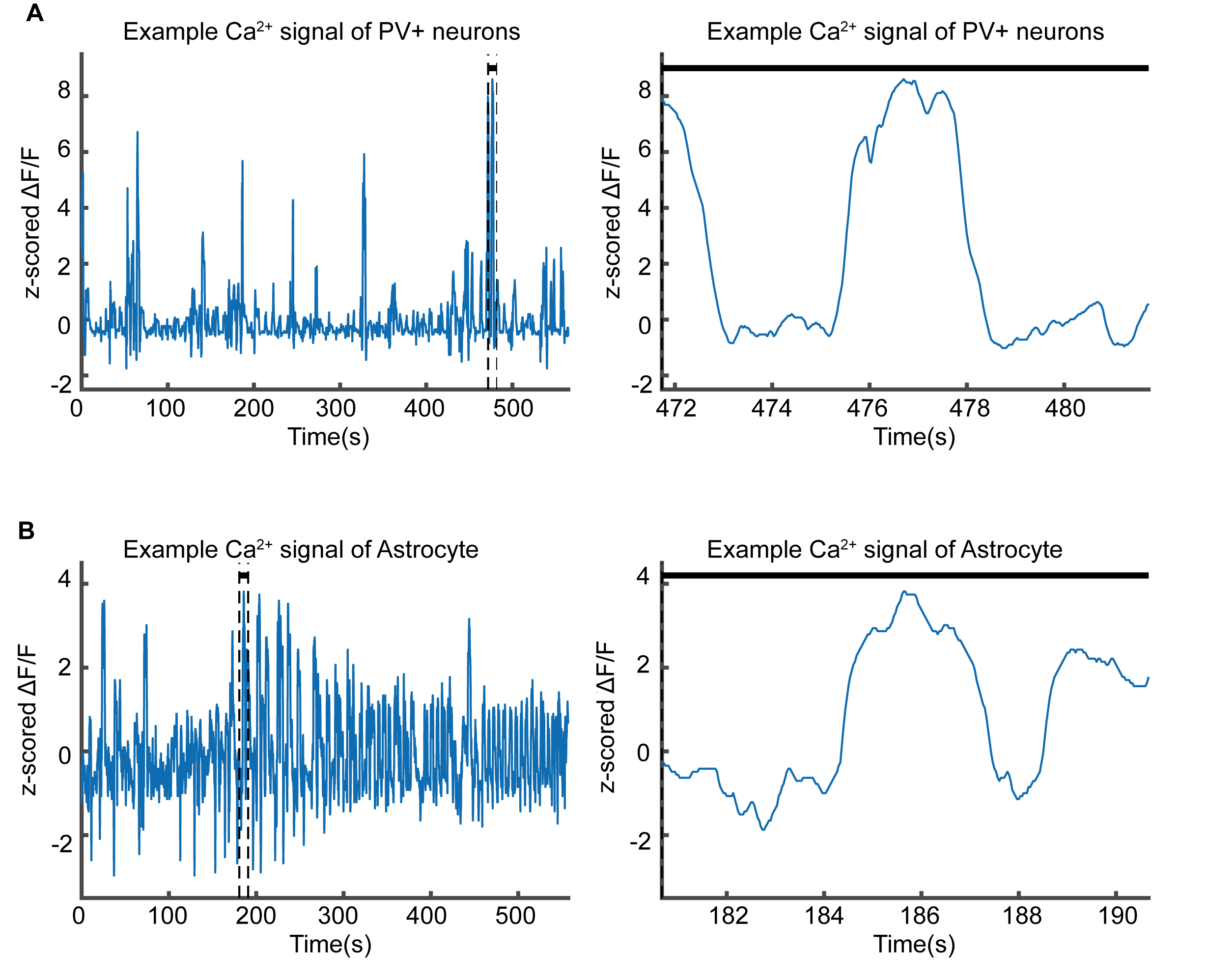

Supplement: Fig 1-2 — Representative Ca2+ signals were recorded from GPe PV + neurons and astrocytes. The left panels show the Ca2+ signal during the entire recording session. The bold black bar on the top of each graph indicates the period zoomed-in on the right panels. The right panels show the Ca2+ signal during the specified period on the left panels. (A) Example Ca2+ signal recorded from PV + neurons. (B) Example Ca2+ signal recorded from astrocytes. Download Fig 1-2, TIF file. [file eneuro-12-ENEURO.0552-24.2025-s003.tif]

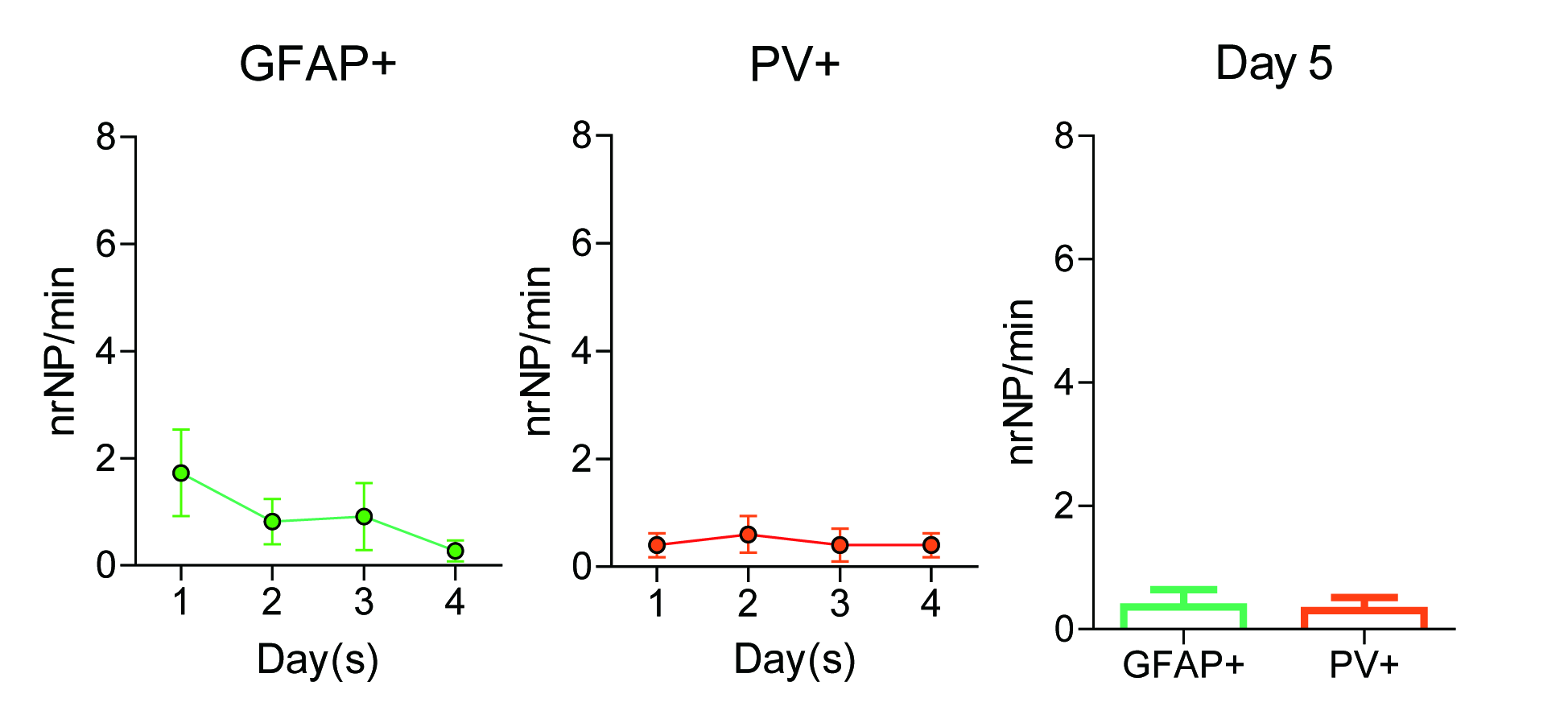

Supplement: Fig 1-3 — Non-rewarded nose-poke behaviors during the FR1 task training and recording sessions. Left and middle for GFAP-Cre mice (n = 11) and PV-Cre mice (n = 10) during the training sessions, respectively. The right panel shows the non-rewarded nose-poke behaviors during the recording session. nrNP/min: the number of non-rewarded nose-pokes per minute. Download Fig 1-3, TIF file. [file eneuro-12-ENEURO.0552-24.2025-s004.tif]

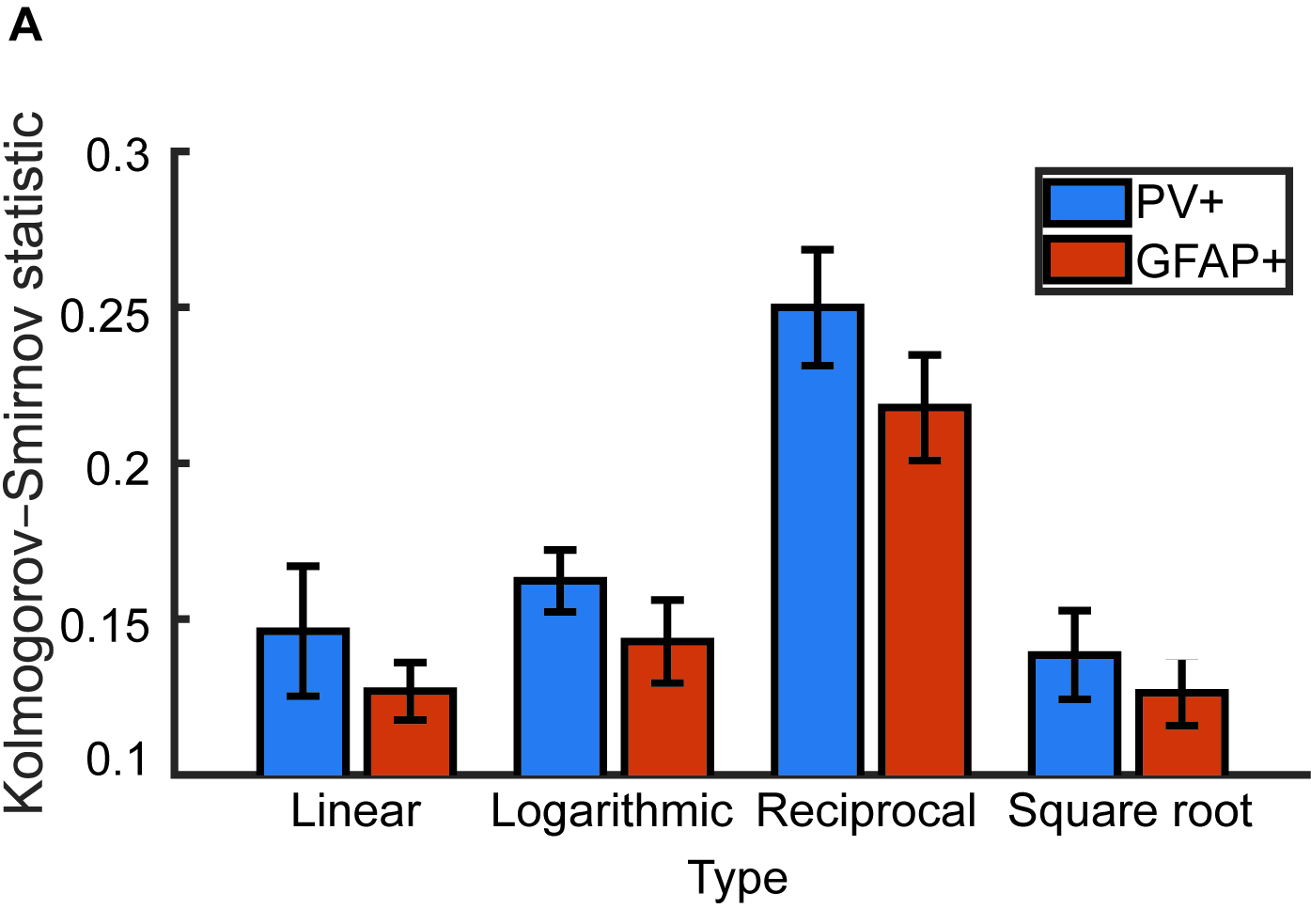

Supplement: Fig 3-1 — Comparison of Kolmogorov-Smirnov statistics across transformation functions. Mixed-effects linear regression, with transformation function type and genotype as fixed factors and subject as a random effect, revealed a statistically significant difference in the K-S statistic between transformation function types, regardless of the genotype (n = 21 mice, including 11 GFAP-Cre mice and 10 PV-Cre mice, P = 3.6429 × 10^−9^ for transformation function type and P = 0.8574 for transformation function type × genotype). Error bars denote mean ± SEM. Download Fig 3-1, TIF file. [file eneuro-12-ENEURO.0552-24.2025-s005.tif]

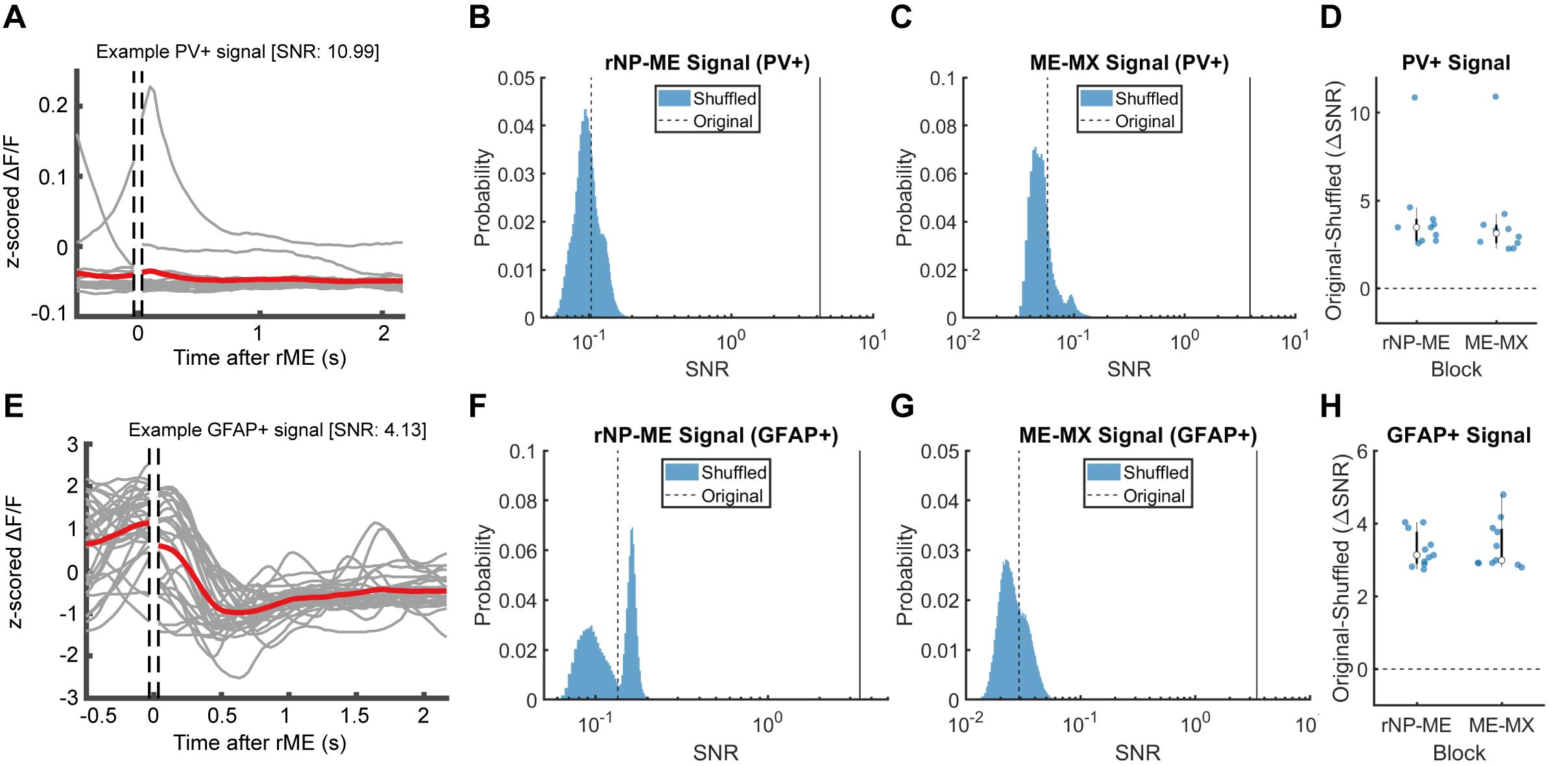

Supplement: Fig 4-1 — Evaluation of Ca2+ signal quality recorded from GPe PV + neurons and astrocytes. (A) Ca2+ signals of GPe PV + neurons from an example PV-Cre mouse. Gray lines represent Ca2+ signals during a single rewarded magazine entry (rME). The thick red line is the average. (B-C) Comparison of SNR values calculated from original and shuffled Ca2+ signals of GPe PV + neurons (n = 310 blocks/rNP-ME and n = 281 blocks/ME-MX from 10 mice). Histograms display the null distribution of shuffled SNR values, with the dotted line indicating the mean of this distribution. The solid line represents the mean of the original SNR values. (B) Comparison during the rNP-ME block. (C) Comparison during the ME-MX block. (D) The individual differences in SNR values between the original and shuffled conditions for PV + neurons. (E) Ca2+ signals of GPe astrocytes from an example GFAP-Cre mouse, with the figure structure identical to (A). (F-G) Comparison of SNR values calculated from original and shuffled Ca2+ signals of GPe astrocytes (n = 467 blocks/rNP-ME and n = 442 blocks/ME-MX from 11 mice), with figure structures identical to (B-C). (F) Comparison during the rNP-ME block. (G) Comparison during the ME-MX block. (H) The individual differences in SNR values between the original and shuffled conditions for astrocytes. Download Fig 4-1, TIF file. [file eneuro-12-ENEURO.0552-24.2025-s006.tif]
